# A real-world pharmacovigilance study of FDA adverse event reporting system (FAERS) events for etrasimod

**DOI:** 10.3389/fphar.2025.1693090

**Published:** 2025-11-11

**Authors:** Yingxiu Wu, Wei Ke, Huibiao Li

**Affiliations:** 1 Foshan Hospital of Traditional Chinese Medicine, Foshan, Guangdong, China; 2 The Eighth Clinical Medical College of Guangzhou University of Chinese Medicine, Foshan, Guangdong, China; 3 The First Affiliated Hospital, Guangzhou University of Chinese Medicine, Guangzhou, China

**Keywords:** etrasimod, FDA adverse event reporting system, disproportionality analyses, pharmacovigilance, adverse event

## Abstract

**Objective:**

To characterize the post-marketing safety profile of etrasimod using the latest data from the FDA Adverse Event Reporting System (FAERS), and to provide a comparative analysis versus other sphingosine-1-phosphate (S1P) receptor modulators.

**Methods:**

AE reports associated with etrasimod were retrieved from FAERS (Q1 2004 – Q2 2025). Disproportionality analyses were conducted using the reporting odds ratio (ROR), proportional reporting ratio (PRR), Bayesian confidence propagation neural network (BCPNN), and multi-item gamma Poisson shrinker (MGPS) methods. A comparative analysis against fingolimod and ozanimod was performed to contextualize findings.

**Results:**

We identified 2,104 AE reports from 967 patients—a larger cohort than previously described. The most frequent AEs were drug ineffectiveness, condition aggravated, headache, and dizziness. Signals were concentrated in gastrointestinal, general, and nervous system disorders. Strong signals included ulcerative proctitis, increased faecal calprotectin, and macular oedema. Critically, our comparative analysis suggested a potentially distinct safety profile for etrasimod, such as a more favorable signal for lymphocyte count decreased compared to other S1P modulators.

**Conclusion:**

This large-scale, updated analysis confirms the established safety profile of etrasimod while providing novel, comparative insights. Our findings, derived from the most recent and extensive FAERS dataset to date, underscore that etrasimod’s real-world safety is characterized by class-related AEs and disease exacerbations. The lack of unexpected signals remains reassuring. The frequent reporting of lack of efficacy highlights the need for close monitoring, and the comparative data offer valuable context for clinicians selecting S1P receptor modulator therapy.

## 1 Introduction

Ulcerative colitis is a chronic, immune-mediated disorder characterized by continuous mucosal inflammation (Ungaro et al., 2017). Current therapeutic strategies aim to relieve symptoms and achieve mucosal healing (Turner et al., 2021); however, a considerable proportion of patients either fail to respond or lose efficacy over time (Singh et al., 2020). Moreover, many conventional treatments require long-term parenteral administration and are associated with risks of infection or malignancy (Agrawal et al., 2020), underscoring the need for safer, orally administered alternatives.

Sphingosine-1-phosphate (S1P) is a bioactive lipid mediator that regulates lymphocyte trafficking through five receptor subtypes (S1P_1_–S1P_5_) (Hla and Brinkmann, 2011). Selective inhibition of S1P_1_ retains lymphocytes in lymphoid tissues, thereby limiting their migration into inflamed intestinal sites (Peyrin-Biroulet et al., 2017; Argollo et al., 2020). Fingolimod, the first S1P receptor modulator, was originally developed for multiple sclerosis. However, its non-selective binding to S1P_2_ and S1P_3_ was associated with pulmonary toxicity, cardiovascular complications, ocular disorders, and malignancies (Jain and Bhatti, 2012; Calabresi et al., 2014; Peyrin-Biroulet et al., 2017). Subsequently, ozanimod—a selective modulator of S1P_1_ and S1P_5_—was approved for both multiple sclerosis and ulcerative colitis (Sandborn et al., 2021). Although the roles of S1P_4_ and S1P_5_ are less well understood, emerging evidence suggests their involvement in dendritic cell trafficking and natural killer cell localization, respectively (Subei and Cohen, 2015; Bryan and Del Poeta, 2018). Compared with fingolimod, ozanimod demonstrates an improved safety profile, though it still requires dose escalation to mitigate first-dose bradycardia (Sandborn et al., 2021) and is prone to food–drug interactions due to its active metabolites (Fda, 2022). These limitations highlight the ongoing need for next-generation S1P receptor modulators with optimized selectivity and tolerability.

Etrasimod (VELSIPITY™) is an orally administered, small-molecule modulator of S1P receptors that was originally developed by Arena Pharmaceuticals and later by Pfizer for the treatment of ulcerative colitis and other immune-mediated inflammatory disorders (Buzard et al., 2014). It selectively targets S1P_1_, S1P_4_, and S1P_5_, with minimal effect on S1P_3_ and no activity on S1P_2_ (Buzard et al., 2014). Modulation of S1P_1_ leads to reversible sequestration of lymphocytes in lymphoid tissues, thereby reducing their circulation (Mendelson et al., 2014). Previous studies have shown that existing S1P modulators, such as fingolimod and ozanimod, are associated with safety concerns due to insufficient receptor selectivity or potent activation of G protein–coupled pathways. In contrast, etrasimod, with its selective activity on S1P_1_, S1P_4_, and S1P_5_ receptors and unique signaling bias, achieves therapeutic efficacy while markedly minimizing related adverse effects (Gaidarov et al., 2025). On 12 October 2023, the US Food and Drug Administration (FDA) approved etrasimod for the treatment of adults with moderately to severely active ulcerative colitis (Shirley, 2024). Although its precise therapeutic mechanism remains incompletely understood, it is generally thought to involve reduced lymphocyte migration into inflamed intestinal mucosa (Siegmund et al., 2023).

Despite its demonstrated therapeutic efficacy, etrasimod use has been associated with adverse events (AEs). Phase II and III trials reported common AEs, including atrioventricular block, anaemia, headache, and disease flares or exacerbations of ulcerative colitis (Sandborn et al., 2020; Sandborn et al., 2023). Therefore, applying data-mining approaches to real-world data is crucial to detect potential safety signals. This is particularly important for newly approved drugs like etrasimod, where post-marketing surveillance is essential to identify rare or long-term adverse events not captured in pre-approval clinical trials.

The FDA Adverse Event Reporting System (FAERS) is a voluntary, spontaneous reporting database that collects post-marketing reports of AEs, product quality issues, and medication errors (Vogel et al., 2020; Setyawan et al., 2021). Given the inherent limitations of clinical trials, such systems play a critical role in pharmacovigilance and signal detection (Lee, 2021). A recent pharmacovigilance study by Guo et al. provided an initial FAERS-based safety profile of etrasimod up to Q4 2024 (Guo et al., 2025). Building on this, our study extends the data to Q2 2025, applies a broader analytical framework with comparative disproportionality analyses against other S1P modulators (fingolimod, ozanimod), and includes sensitivity analyses. These updates enhance the robustness of signal detection and offer a more comprehensive, real-world assessment of etrasimod’s post-marketing safety.

## 2 Methods and materials

### 2.1 Data source

Data were obtained from the FAERS database (https://fis.fda.gov/extensions/FPD-QDE-FAERS/FPD-QDE-FAERS.html). The search covered the period from the first quarter of 2004 to the second quarter of 2025, using “ETRASIMOD” as the keyword and including reports in which it was designated as the primary suspected drug. The FAERS database comprises seven datasets: demographic information, drug information, AEs, patient outcomes, report sources, therapy details, and drug indications. All were used in this study. Statistical analyses were performed with SAS version 9.4, one of the software tools recommended by the FDA for FAERS data mining.

### 2.2 Data cleaning

As FAERS relies on spontaneous reporting, the database may contain duplicate records or withdrawn/deleted reports. The FDA provides guidelines for deduplication as well as lists of deleted reports, which were strictly followed in this study. Specifically, deduplication was conducted using the DEMO table variables PRIMARYID, CASEID, and FDA_DT, sorted by CASEID, FDA_DT, and PRIMARYID. For identical CASEIDs, the record with the most recent FDA_DT was retained; if both CASEID and FDA_DT were identical, the record with the largest PRIMARYID was kept. Additionally, since 2019, each quarterly dataset has included a list of deleted reports, which were further removed according to the corresponding CASEIDs.

### 2.3 Use of the MedDRA dictionary

AEs were standardized and coded using the Medical Dictionary for Regulatory Activities (MedDRA, version 27.0). In FAERS, AEs are coded by Preferred Terms (PT), which are grouped into System Organ Class (SOC). Because MedDRA is updated biannually (March and September), the most recent version was applied to ensure consistency in PT and SOC classification for subsequent analyses.

### 2.4 Statistical analysis

Descriptive statistics were used to summarize the characteristics of etrasimod-related AE reports. Signal detection was performed using four disproportionality algorithms: reporting odds ratio (ROR) (Sakaeda et al., 2013), proportional reporting ratio (PRR) (Kelly et al., 2007), Bayesian confidence propagation neural network (BCPNN) (Bate et al., 1998), and multinomial gamma Poisson shrinkage (MGPS) (Sakaeda et al., 2013). ROR and PRR, as frequency-based methods, are sensitive but less specific, whereas BCPNN and MGPS, as Bayesian approaches, handle complex data structures yet with relatively lower sensitivity (Zou et al., 2023). To enhance robustness, multiple algorithms were combined. Higher values of these parameters reflect stronger signal intensity, serving as quantitative indicators. The formulas and criteria are summarized in Tables 1, 2 (Liu et al., 2024; Tang et al., 2024).

**TABLE 1:** Ratio imbalance measurement algorithm.

Drug	Number of target adverse event reports	Number of other adverse event reports	Total
Target drug	a	b	a + b
Other drug	c	d	c + d
Total	a + c	b + d	N = a + b + c + d

**TABLE 2:** Calculation formula and standard of signal detection.

Method	Formula	Threshold value
ROR	$PRR=\frac{a/\left(a+b\right)}{c/\left(c+d\right)}$ $SE\left(lnPRR\right)=\sqrt{\frac{1}{a}-\frac{1}{a+b}+\frac{1}{c}-\frac{1}{c+d}}$ $95\%CI=e^{\ln\left(PRR\right)\pm 1.96\sqrt{\frac{1}{a}-\frac{1}{a+b}+\frac{1}{c}-\frac{1}{c+d}}}$ $x^{2}=\frac{{\left(ad-bc\right)}^{2}\left(a+b+c+d\right)}{\left(a+b\right)\left(a+c\right)\left(c+d\right)\left(b+\right)}$	If a ≥3 and the lower limit of the 95% CI >1, then one signal is generated
PRR	$PRR=\frac{a/\left(a+b\right)}{c/\left(c+d\right)}$ $SE\left(lnPRR\right)=\sqrt{\frac{1}{a}-\frac{1}{a+b}+\frac{1}{c}-\frac{1}{c+d}}$ $95\%CI=e^{\ln\left(PRR\right)\pm 1.96\sqrt{\frac{1}{a}-\frac{1}{a+b}+\frac{1}{c}-\frac{1}{c+d}}}$ $x^{2}=\frac{{\left(ad-bc\right)}^{2}\left(a+b+c+d\right)}{\left(a+b\right)\left(a+c\right)\left(c+d\right)\left(b+d\right)}$	PRR lower limit of confidence interval method: If a ≥3 and the lower limit of the 95% CI >1, then one signal is generated MHRA comprehensive criteria: If a ≥3, PRR ≥2, and χ^2^ ≥ 4, then one signal is generated
BCPNN	$IC=\log_{2}\frac{p\left(x,y\right)}{p\left(x\right)p\left(y\right)}=\log_{2}\frac{a\left(a+b+c+d\right)}{\left(a+b\right)\left(a+c\right)}$ $E\left(IC\right)=\log_{2}\frac{\left(a+\gamma 11\right)\left(a+b+c+d+\alpha \right)\left(a+b+c+d+\beta \right)}{\left(a+b+c+d+\gamma \right)\left(a+b+\alpha 1\right)\left(a+c+\beta 1\right)}$ $V\left(IC\right)=\frac{1}{{\left(\ln2\right)}^{2}}\left\{\left[\frac{\left(a+b+c+d\right)-a+\gamma -\gamma 11}{\left(a+\gamma 11\right)\left(1+a+b+c+d+\gamma \right)}\right]+\left[\frac{\left(a+b+c+d\right)-\left(a+b\right)+\alpha -\alpha 1}{\left(a+b+\alpha 1\right)\left(1+a+b+c+d+\alpha \right)}\right]+\left[\frac{\left(a+b+c+d\right)-\left(a+c\right)+\beta -\beta 1}{\left(a+c+\beta 1\right)\left(1+a+b+c+d+\beta \right)}\right]\right\}$ $\gamma =\gamma 11\frac{\left(a+b+c+d+\alpha \right)\left(a+b+c+d+\beta \right)}{\left(a+b+\alpha 1\right)\left(a+c+\beta 1\right)}$ $IC-2SD=E\left(IC\right)-2\sqrt{V\left(IC\right)}$ α1 = β1 = 1; α = β = 2; γ11 = 1	If the lower limit of the confidence interval (IC – 2SD) is greater than 0, then one signal is generated
MGPS	$EBGM=\frac{a\left(a+b+c+d\right)}{\left(a+c\right)\left(a+b\right)}$ $95\%CI=e^{\ln\left(EBGM\right)\pm 1.96\sqrt{\left(\frac{1}{a}+\frac{1}{b}+\frac{1}{c}+\frac{1}{d}\right)}}$	If EBGM05 >2, then one signal is generated EBGM05 represents the lower limit of the 95% confidence interval of EBGM.

## 3 Results

### 3.1 General overview of adverse event reports related to etrasimod

**FIGURE 1:**
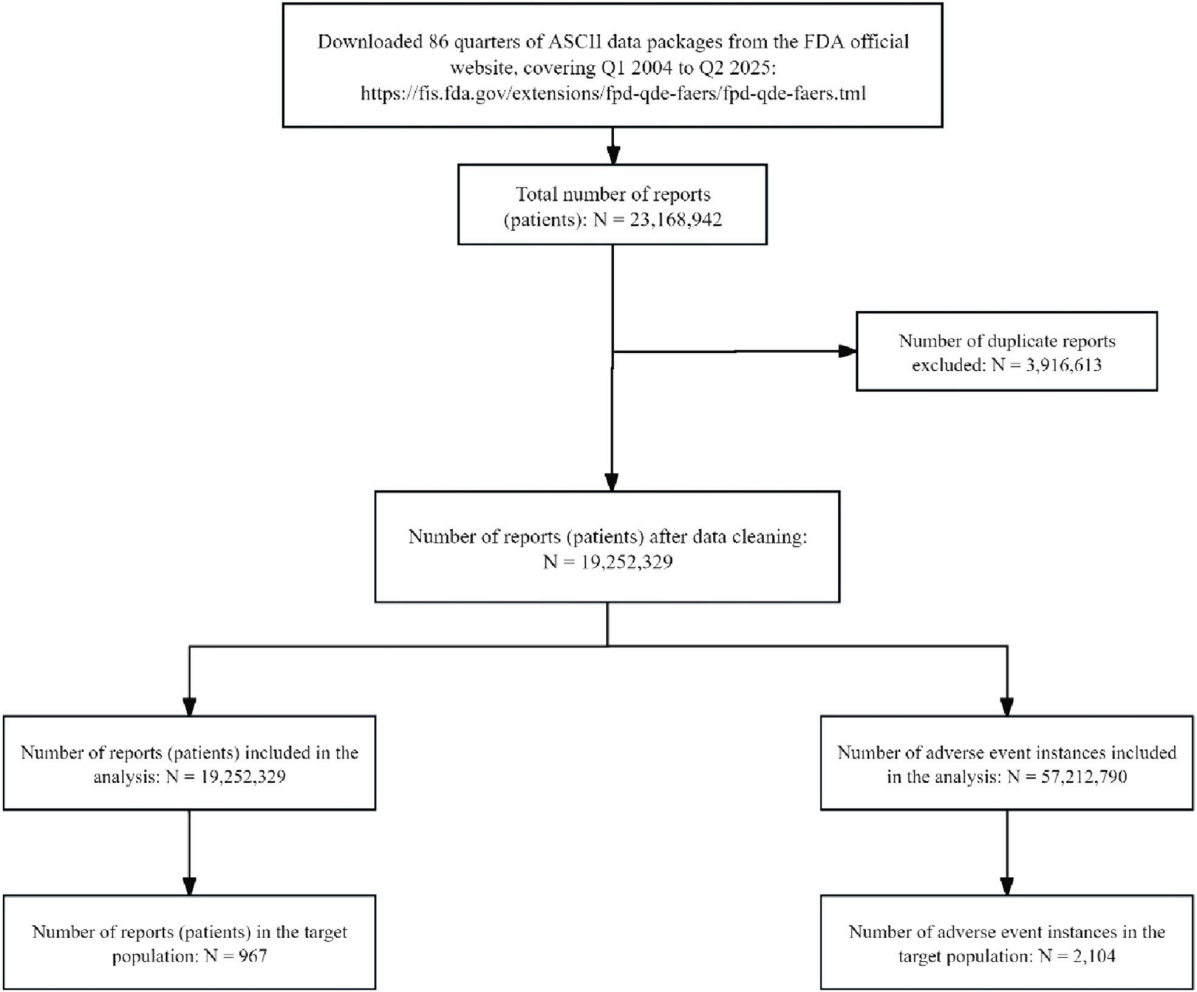
Flowchart of identifying AE cases of etrasimod from the FAERS database.

From the first quarter of 2004 to the second quarter of 2025, a total of 86 quarterly datasets were included. Overall, 19,252,329 background patients were identified, accounting for 57,212,790 AE records. Among them, 967 patients were exposed to etrasimod, with 2,104 AEs reported, as shown in Figure 1.

The proportions of female (46.95%) and male (42.40%) patients were comparable. Young adults (18–44 years) constituted the largest group experiencing AEs (35.37%). No cases were recorded prior to 2023, but a marked increase in reporting occurred in 2024 and 2025.

Consumers accounted for nearly half of all reports (47.78%), followed by physicians (28.34%) and pharmacists (23.78%). Geographically, most reports originated from North America (98.24%), with the United States contributing the vast majority (94.31%). Detailed demographic and clinical characteristics are presented in Table 3.

**TABLE 3:** Demographic characteristics of patients.

Characteristics	Case number, n	Case proportion, %
Gender		
Female	454	46.95
Male	410	42.40
Not specified	103	10.65
Age		
<18	5	0.52
18–44	342	35.37
45–64	287	29.68
≥65	122	12.62
Not specified	211	21.82
Age (quantitative)		
N (Missing)	756 (211)	—
Mean (SD)	46.41 (16.26)	—
Median (Q1,Q3)	47.00 (34.00,59.00)	—
Min, Max	0.42,87.00	—
Reporting year		
2023	9	0.93
2024	510	52.74
2025	448	46.33
Reporter		
Consumer	462	47.78
Not specified	1	0.10
Pharmacist	230	23.78
Physician	274	28.34
Reported countries (top five)		
United States of America	913	94.42
Canada	33	3.41
Puerto Rico	5	0.52
Australia	4	0.41
Austria	2	0.21
outcome^a^		
Life-threatening	1	0.10
Hospitalization - initial or prolonged	61	6.31
Disability	2	0.21
Death	2	0.21
Congenital anomaly	0	0.00
Required intervention to prevent permanent impairment/damage	0	0.00
Other	246	25.44
Time from drug administration to adverse event (days, categorical)^b^		
0–30d	42	4.34
31–60d	9	0.93
61–90d	9	0.93
91–120d	1	0.10
121–150d	1	0.10
151–180d	1	0.10
181–360d	4	0.41
>360d	1	0.10
Missing or abnormal values (<0)	899	92.97
Time from drug administration to adverse event (days, values <0 set to missing)^c^		
N (Missing)	68 (899)	—
Mean (SD)	45.68 (74.65)	—
Median (Q1, Q3)	17.00 (1.00,56.50)	—
Min, Max	0.00,362.00	—
Weight		
N (Missing)	65 (902)	—
Mean (SD)	73.24 (18.78)	—
Median (Q1, Q3)	72.57 (60.00,81.65)	—
Min, Max	42.18,147.42	—

^a^ Outcomes are defined at the patient level rather than at the specific adverse event level. A single patient may have multiple outcomes; therefore, percentages may not sum to 100%.

^b^ The adverse event date refers to the first occurrence of any adverse event for a patient (not tied to a specific event).

^c^ For calculation of time (days) from drug administration to adverse event, negative values were treated as missing before statistical analysis. For this type of data, the median is generally more informative than the mean.

### 3.2 Signal of system organ class

At the System Organ Class (SOC) level, the signal strength of Etrasimod-associated adverse events (AEs) is summarized in Table 4. Statistical analysis indicated that Etrasimod was associated with AEs across 25 organ systems. The most frequently reported SOCs included General disorders and administration site conditions (598 cases, 28.42%), Gastrointestinal disorders (422 cases, 20.06%), Nervous system disorders (195 cases, 9.27%), Investigations (176 cases, 8.37%), and Infections and infestations (98 cases, 4.66%).

**TABLE 4:** Distribution of target drug–related adverse events by SOC.

System organ class (SOC)	Case number, n	Case proportion, %
General disorders and administration site conditions	598	28.42
Gastrointestinal disorders	422	20.06
Nervous system disorders	195	9.27
Investigations	176	8.37
Infections and infestations	98	4.66
Eye disorders	86	4.09
Skin and subcutaneous tissue disorders	79	3.75
Musculoskeletal and connective tissue disorders	68	3.23
Injury, poisoning and procedural complications	63	2.99
Cardiac disorders	51	2.42
Respiratory, thoracic and mediastinal disorders	43	2.04
Psychiatric disorders	42	2
Vascular disorders	40	1.9
Immune system disorders	31	1.47
Metabolism and nutrition disorders	26	1.24
Blood and lymphatic system disorders	20	0.95
Renal and urinary disorders	14	0.67
Surgical and medical procedures	12	0.57
Hepatobiliary disorders	10	0.48
Neoplasms benign, malignant and unspecified (incl cysts and polyps)	9	0.43
Ear and labyrinth disorders	6	0.29
Reproductive system and breast disorders	6	0.29
Endocrine disorders	5	0.24
Social circumstances	3	0.14
Congenital, familial and genetic disorders	1	0.05

Cases represent the number of adverse events classified under each System Organ Class (SOC).

Proportion (%) is calculated as the number of adverse events within each SOC divided by the total number of adverse events.

**FIGURE 2:**
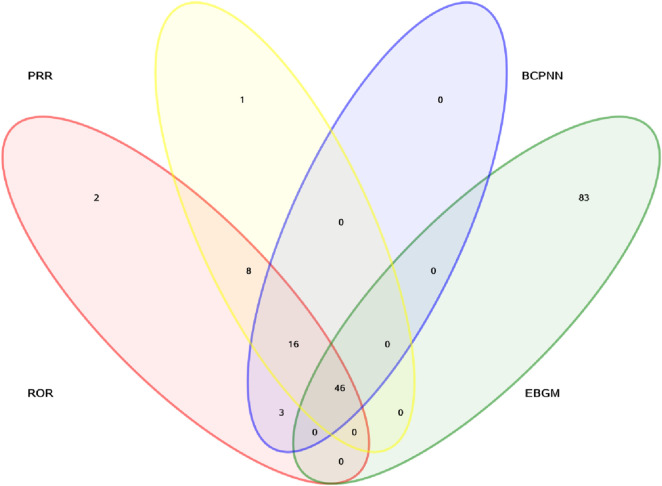
The Venn diagram of ROR, PRR, BCPNN, and MGPS.

In addition, signal detection was performed by combining four methods—ROR, PRR, BCPNN, and MGPS—with the following threshold criteria: a ≥3, the lower limit of the 95% CI for ROR >1, PRR ≥2 with a chi-square value ≥4, IC-2SD >0, and EBGM05 >2. Based on these criteria, a total of 46 signals involving different organ systems were identified, as shown in Figure 2 and Table 5.

**TABLE 5:** Distribution of positive signals of adverse events of the target drug across different System Organ Classes (SOC).

System organ class (SOC)	Case number, n	Case proportion, %
General disorders and administration site conditions	8	17.39
Gastrointestinal disorders	15	32.61
Nervous system disorders	3	6.52
Investigations	8	17.39
Infections and infestations	2	4.35
Eye disorders	3	6.52
Skin and subcutaneous tissue disorders	0	0
Musculoskeletal and connective tissue disorders	0	0
Injury, poisoning and procedural complications	0	0
Cardiac disorders	1	2.17
Respiratory, thoracic and mediastinal disorders	0	0
Psychiatric disorders	3	6.52
Vascular disorders	0	0
Immune system disorders	1	2.17
Metabolism and nutrition disorders	0	0
Blood and lymphatic system disorders	1	2.17
Renal and urinary disorders	0	0
Surgical and medical procedures	0	0
Hepatobiliary disorders	0	0
Neoplasms benign, malignant and unspecified (incl cysts and polyps)	0	0
Ear and labyrinth disorders	0	0
Reproductive system and breast disorders	0	0
Endocrine disorders	1	2.17
Social circumstances	0	0
Congenital, familial and genetic disorders	0	0
Product issues	0	0
Pregnancy, puerperium and perinatal conditions	0	0
Total	46	100

The number of positive signals represents the count of Preferred Terms (PTs) within each SOC that were identified as signals using the specified method. This refers to the number of distinct PTs, not the number of PT occurrences.

The proportion (%) is calculated as the number of signals within each SOC divided by the total number of signals detected for the target drug.

### 3.3 Signal of preferred terms

**FIGURE 3:**
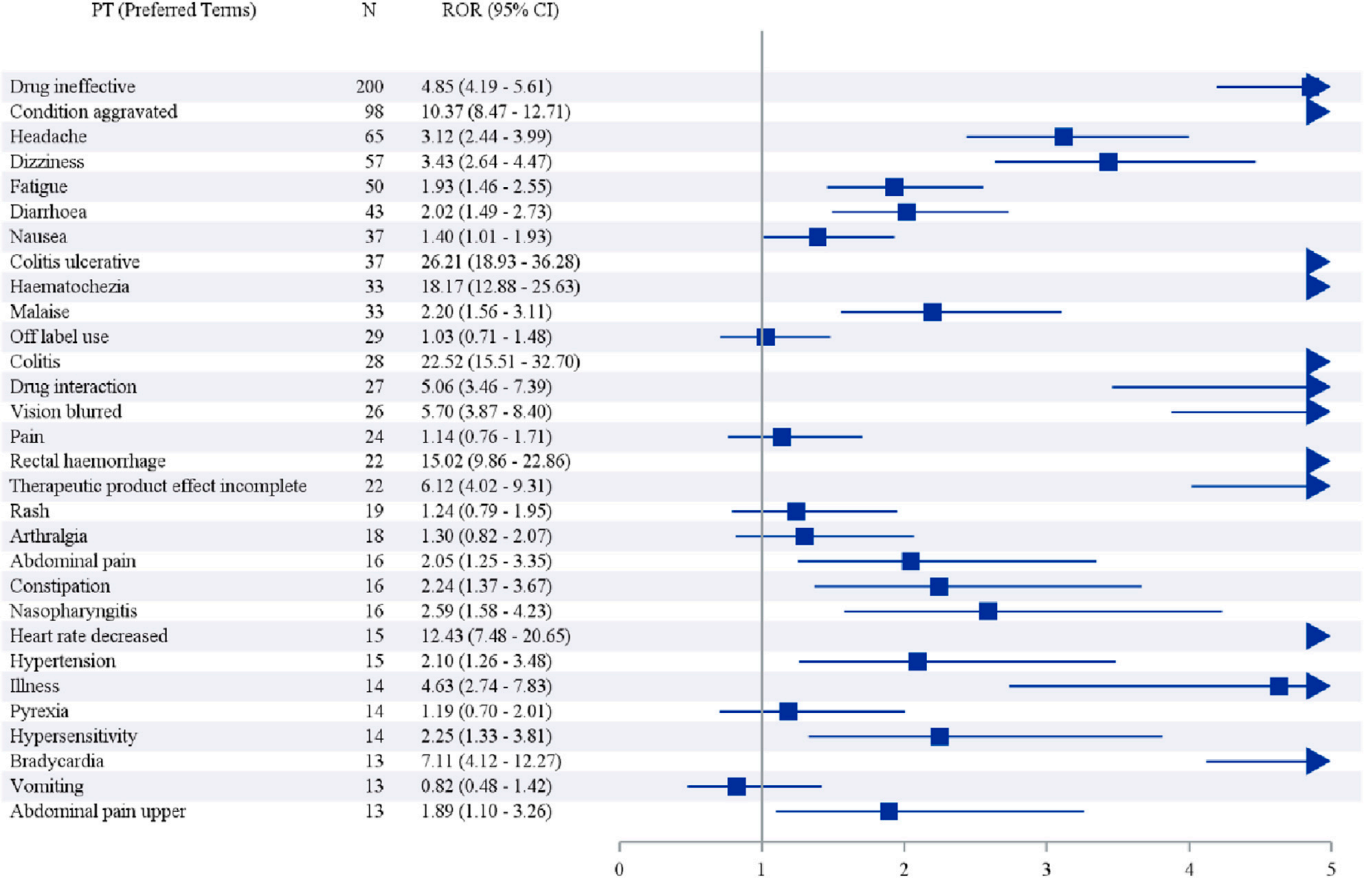
Forest plot for the frequency of the target drug.

We further examined the PT signals and identified 30 preferred terms (PTs) that showed significant disproportionality across all four algorithms (Table 6; Figure 3). The most frequently reported events included Drug ineffective (200 cases), Condition aggravated (98 cases), Headache (65 cases), Dizziness (57 cases), and Fatigue (50 cases).

**TABLE 6:** Top 30 Preferred Terms (PTs) ranked by frequency for the target drug.

Preferred terms	Case number, n	ROR (95% CI)	PRR (Chi-square)	IC (IC-2SD)	EBGM (EBGM05)
Drug ineffective	200	4.85 (4.19–5.61)	4.48 (553.09)	2.16 (1.93)	4.48 (3.88)
Condition aggravated	98	10.37 (8.47–12.71)	9.94 (791.09)	3.31 (2.89)	9.93 (8.11)
Headache	65	3.12 (2.44–3.99)	3.05 (90.75)	1.61 (1.21)	3.05 (2.39)
Dizziness	57	3.43 (2.64–4.47)	3.37 (95.59)	1.75 (1.31)	3.37 (2.59)
Fatigue	50	1.93 (1.46–2.55)	1.91 (21.85)	0.93 (0.50)	1.91 (1.44)
Diarrhoea	43	2.02 (1.49–2.73)	2.00 (21.61)	1.00 (0.53)	2.00 (1.48)
Nausea	37	1.40 (1.01–1.93)	1.39 (4.08)	0.47 (-0.01)	1.39 (1.00)
Colitis ulcerative	37	26.21 (18.93–36.28)	25.77 (880.54)	4.69 (3.49)	25.74 (18.60)
Haematochezia	33	18.17 (12.88–25.63)	17.90 (526.60)	4.16 (3.08)	17.89 (12.68)
Malaise	33	2.20 (1.56–3.11)	2.18 (21.31)	1.13 (0.58)	2.18 (1.55)
Off label use	29	1.03 (0.71–1.48)	1.03 (0.02)	0.04 (-0.49)	1.03 (0.71)
Colitis	28	22.52 (15.51–32.70)	22.23 (567.62)	4.47 (3.14)	22.21 (15.30)
Drug interaction	27	5.06 (3.46–7.39)	5.01 (86.77)	2.32 (1.58)	5.01 (3.42)
Vision blurred	26	5.70 (3.87–8.40)	5.65 (99.60)	2.50 (1.71)	5.65 (3.83)
Pain	24	1.14 (0.76–1.71)	1.14 (0.42)	0.19 (-0.40)	1.14 (0.76)
Rectal haemorrhage	22	15.02 (9.86–22.86)	14.87 (284.63)	3.89 (2.61)	14.86 (9.76)
Therapeutic product effect incomplete	22	6.12 (4.02–9.31)	6.06 (93.15)	2.60 (1.71)	6.06 (3.98)
Rash	19	1.24 (0.79–1.95)	1.24 (0.89)	0.31 (-0.36)	1.24 (0.79)
Arthralgia	18	1.30 (0.82–2.07)	1.30 (1.23)	0.37 (-0.31)	1.30 (0.82)
Abdominal pain	16	2.05 (1.25–3.35)	2.04 (8.51)	1.03 (0.24)	2.04 (1.25)
Constipation	16	2.24 (1.37–3.67)	2.24 (10.96)	1.16 (0.36)	2.24 (1.37)
Nasopharyngitis	16	2.59 (1.58–4.23)	2.58 (15.47)	1.37 (0.53)	2.58 (1.58)
Heart rate decreased	15	12.43 (7.48–20.65)	12.35 (156.42)	3.63 (2.13)	12.34 (7.43)
Hypertension	15	2.10 (1.26–3.48)	2.09 (8.55)	1.06 (0.24)	2.09 (1.26)
Illness	14	4.63 (2.74–7.83)	4.61 (39.59)	2.20 (1.14)	4.61 (2.72)
Pyrexia	14	1.19 (0.70–2.01)	1.19 (0.41)	0.25 (-0.52)	1.19 (0.70)
Hypersensitivity	14	2.25 (1.33–3.81)	2.24 (9.68)	1.17 (0.30)	2.24 (1.33)
Bradycardia	13	7.11 (4.12–12.27)	7.08 (67.88)	2.82 (1.53)	7.08 (4.10)
Vomiting	13	0.82 (0.48–1.42)	0.82 (0.49)	−0.28 (-1.03)	0.82 (0.48)
Abdominal pain upper	13	1.89 (1.10–3.26)	1.89 (5.43)	0.92 (0.05)	1.89 (1.09)

This table is ranked by frequency and presents only positive-signal Preferred Terms (PTs).

If the number of positive-signal PTs is fewer than 30, all detected PTs are presented.

**FIGURE 4:**
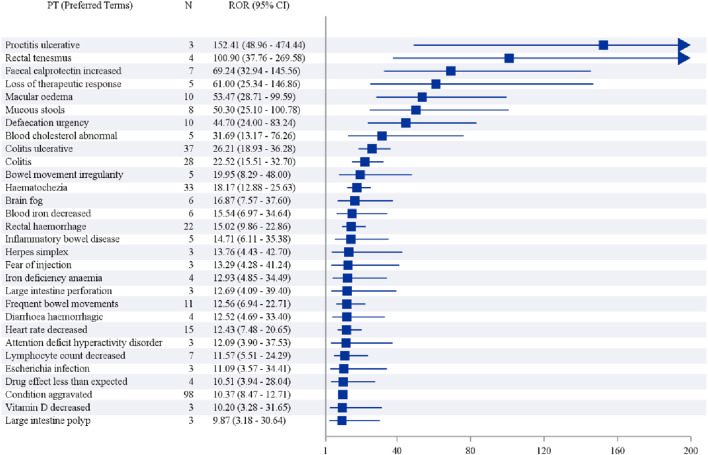
Forest plot for the signal strength for the target drug.

In addition, when ranked by the strength of positive signals, the leading PTs were Proctitis ulcerative (3 cases), Rectal tenesmus (4 cases), Faecal calprotectin increased (7 cases), Loss of therapeutic response (5 cases), and Macular oedema (10 cases), as shown in Table 7 and Figure 4.

**TABLE 7:** Top 30 Preferred Terms (PTs) ranked by signal strength for the target drug.

Preferred terms	Case number, n	ROR (95% CI)	PRR (Chi-square)	IC (IC-2SD)	EBGM (EBGM05)
Proctitis ulcerative	3	152.41 (48.96–474.44)	152.19 (448.09)	7.24 (0.52)	151.35 (48.62)
Rectal tenesmus	4	100.90 (37.76–269.58)	100.71 (393.43)	6.65 (0.97)	100.34 (37.56)
Faecal calprotectin increased	7	69.24 (32.94–145.56)	69.01 (468.03)	6.11 (1.84)	68.84 (32.75)
Loss of therapeutic response	5	61.00 (25.34–146.86)	60.86 (293.73)	5.92 (1.29)	60.72 (25.22)
Macular oedema	10	53.47 (28.71–99.59)	53.22 (511.46)	5.73 (2.34)	53.12 (28.52)
Mucous stools	8	50.30 (25.10–100.78)	50.11 (384.37)	5.64 (1.99)	50.02 (24.97)
Defaecation urgency	10	44.70 (24.00–83.24)	44.49 (424.46)	5.47 (2.29)	44.42 (23.85)
Blood cholesterol abnormal	5	31.69 (13.17–76.26)	31.62 (148.09)	4.98 (1.19)	31.58 (13.12)
Colitis ulcerative	37	26.21 (18.93–36.28)	25.77 (880.54)	4.69 (3.49)	25.74 (18.60)
Colitis	28	22.52 (15.51–32.70)	22.23 (567.62)	4.47 (3.14)	22.21 (15.30)
Bowel movement irregularity	5	19.95 (8.29–48.00)	19.91 (89.73)	4.31 (1.08)	19.89 (8.27)
Haematochezia	33	18.17 (12.88–25.63)	17.90 (526.60)	4.16 (3.08)	17.89 (12.68)
Brain fog	6	16.87 (7.57–37.60)	16.82 (89.26)	4.07 (1.27)	16.81 (7.54)
Blood iron decreased	6	15.54 (6.97–34.64)	15.50 (81.34)	3.95 (1.24)	15.49 (6.95)
Rectal haemorrhage	22	15.02 (9.86–22.86)	14.87 (284.63)	3.89 (2.61)	14.86 (9.76)
Inflammatory bowel disease	5	14.71 (6.11–35.38)	14.67 (63.68)	3.87 (0.98)	14.67 (6.10)
Herpes simplex	3	13.76 (4.43–42.70)	13.74 (35.42)	3.78 (0.27)	13.73 (4.42)
Fear of injection	3	13.29 (4.28–41.24)	13.27 (34.02)	3.73 (0.26)	13.26 (4.27)
Iron deficiency anaemia	4	12.93 (4.85–34.49)	12.91 (43.93)	3.69 (0.64)	12.90 (4.84)
Large intestine perforation	3	12.69 (4.09–39.40)	12.68 (32.26)	3.66 (0.25)	12.67 (4.08)
Frequent bowel movements	11	12.56 (6.94–22.71)	12.50 (116.33)	3.64 (1.84)	12.49 (6.91)
Diarrhoea haemorrhagic	4	12.52 (4.69–33.40)	12.50 (42.30)	3.64 (0.63)	12.49 (4.68)
Heart rate decreased	15	12.43 (7.48–20.65)	12.35 (156.42)	3.63 (2.13)	12.34 (7.43)
Attention deficit hyperactivity disorder	3	12.09 (3.90–37.53)	12.07 (30.46)	3.59 (0.24)	12.07 (3.89)
Lymphocyte count decreased	7	11.57 (5.51–24.29)	11.53 (67.31)	3.53 (1.29)	11.53 (5.49)
*Escherichia* infection	3	11.09 (3.57–34.41)	11.07 (27.48)	3.47 (0.21)	11.07 (3.57)
Drug effect less than expected	4	10.51 (3.94–28.04)	10.50 (34.36)	3.39 (0.56)	10.49 (3.93)
Condition aggravated	98	10.37 (8.47–12.71)	9.94 (791.09)	3.31 (2.89)	9.93 (8.11)
Vitamin D decreased	3	10.20 (3.28–31.65)	10.18 (24.84)	3.35 (0.18)	10.18 (3.28)
Large intestine polyp	3	9.87 (3.18–30.64)	9.86 (23.87)	3.30 (0.17)	9.85 (3.18)

This table is ranked by frequency and presents only positive-signal Preferred Terms (PTs).

If the number of positive-signal PTs is fewer than 30, all detected PTs are presented.

### 3.4 Sensitivity analysis and disproportionality analysis

Given the complexity of UC and the frequent use of multiple medications in its treatment, a sensitivity analysis was conducted by including all reports in which Etrasimod was listed under any drug role (e.g., “primary suspect,” “secondary suspect,” or “concomitant”). The results showed that, when the analysis was not restricted by the level of suspicion, the most frequently reported adverse events were drug ineffective (n = 212), condition aggravated (n = 99), headache (n = 65), dizziness (n = 58), and fatigue (n = 57). Regarding positive signals, the strongest ones were adenosine deaminase decreased (n = 4), large intestine erosion (n = 7), ileocaecal resection (n = 4), ileal ulcer (n = 8), and proctitis ulcerative (n = 3) (Supplementary Figures S1–S3; Supplementary Tables S1–S4).

These findings indicate that while the pattern of the most common events showed little variation, the strength of the positive signals differed substantially, suggesting that when Etrasimod is identified as the primary suspect drug, the analysis demonstrates greater sensitivity.

In addition, a comparative disproportionality analysis was performed to evaluate the signal strength of key adverse events associated with Etrasimod in comparison with other S1P modulators, including Fingolimod and Ozanimod (Supplementary Table S5). The results showed that, based on direct comparisons within the same FAERS database, Etrasimod exhibited a relatively favorable safety profile for certain adverse events, such as lymphocyte count decreased. However, no clear advantage was observed for other key adverse events. This may be attributed to the relatively recent market introduction of Etrasimod and the limited number of available reports, warranting further post-marketing surveillance and verification.

## 4 Discussion

This FAERS-based pharmacovigilance analysis provides a comprehensive real-world assessment of the post-marketing safety profile of etrasimod. The most frequently reported AEs were drug ineffectiveness and condition aggravated, suggesting that a notable proportion of patients experienced insufficient therapeutic benefit or disease exacerbation despite treatment. Other commonly reported AEs, such as headache, dizziness, and fatigue, were generally mild tolerability-related events and are consistent with observations from clinical trials (Lees et al., 2024). In total, PT signals spanning multiple organ systems, with a predominance in gastrointestinal disorders, general disorders and administration site conditions, investigations, nervous system disorders, and infections and infestations.

Certain AE (PTs) exhibited relatively strong signal strength. For example, “ulcerative proctitis,” although reported in a relatively small number of cases, still showed a high disproportionality score. However, it should be noted that certain PT terms, such as ulcerative proctitis, large intestine polyps, and herpes simplex, should not be regarded as drug-related adverse reactions. These terms represent distinct disease entities that are not inherently caused by drug exposure and therefore do not fall within the scope of adverse drug reactions. Moreover, patients are unlikely to directly report such terms, as they may not be familiar with the corresponding medical terminology. Therefore, in clinical practice, physicians, nurses, and pharmacists should be aware that these conditions do not need to be reported as drug-related adverse events. These signals, albeit reported in only a few patients, underscore that disease flares or localized inflammatory exacerbations can occur during Etrasimod therapy. They may reflect either primary non-response, secondary loss of response over time, or possibly rebound disease activity in cases of treatment interruption. In line with these, we observed “Faecal calprotectin increased” as a notable signal; faecal calprotectin is a biomarker of intestinal inflammation, and its elevation in Etrasimod-treated patients provides objective corroboration of recurrent disease activity. On the other hand, several class-effect signals were also prominent. “Heart rate decreased” (often reported alongside “bradycardia”) was a significant cardiovascular signal, consistent with the known S1P_1 receptor-mediated negative chronotropic effect of this drug class (Vermeire et al., 2025). In fact, first-dose bradycardia is a recognized pharmacodynamic effect of S1P modulators, and cases of transient sinus bradycardia or atrioventricular block have been documented with Etrasimod (Vermeire et al., 2025). An “Eye disorders” signal of interest was “Macular oedema,” an adverse effect well-described with first-generation S1P modulators like fingolimod (Chen et al., 2025). While only a small number of macular edema cases appeared in FAERS, its presence is notable given that S1P receptor modulation in ocular tissues can increase vascular permeability. We also detected signals such as “Blood cholesterol abnormal,” hinting at metabolic changes; interestingly, fingolimod and other S1P modulators have been reported to cause modest lipid profile alterations (Chen et al., 2025). Although the clinical significance of a cholesterol signal with Etrasimod is unclear, it suggests a need to further monitor metabolic parameters in long-term use. Overall, the PT signals with the highest reporting odds either reflected worsening of the underlying disease (e.g., colitis-related terms) or mechanistic effects of S1P_1 modulation (cardiac and ophthalmic effects), reinforcing the biologic plausibility of these safety findings.

Many of these safety signals can be directly understood in the context of Etrasimod’s mechanism of action and pharmacology. Etrasimod is a selective modulator of S1P receptors 1, 4, and 5, with negligible activity at S1P_3 and none at S1P_2. By functionally antagonizing S1P_1 on lymphocytes, Etrasimod causes a reversible sequestration of lymphocytes in lymphoid organs, thereby reducing peripheral circulation of T and B cells. This immune modulation is therapeutically beneficial in ulcerative colitis but also underlies the drug’s immunosuppressive adverse effects. Consistent with this, our findings of infection-related signals (e.g., upper respiratory infections like nasopharyngitis) are expected class effects; S1P modulators including Etrasimod have been associated with increased susceptibility to infections due to lymphopenia (Sandborn et al., 2023). The cardiac signals observed (bradycardia and heart rate decreases) are directly attributable to S1P_1 receptor engagement on atrial myocytes and the cardiac conduction system. S1P_1 modulation can activate the G_i protein pathway in the sinoatrial node, leading to a slowing of heart rate. Indeed, Etrasimod’s trials reported mild, transient bradycardia predominantly on day 1 of dosing (Vermeire et al., 2025). While Etrasimod’s greater selectivity (sparing S1P_3) might theoretically reduce this risk, the FAERS reports (and at least one case in phase III trials) show that clinicians must remain vigilant for visual symptoms (Dubinsky et al., 2025). Finally, the cluster of signals related to “drug ineffective”, “condition aggravated”, and “therapeutic response lost” highlight an important consideration in real-world use: some patients will experience lack of efficacy or disease rebound. This could stem from the heterogeneous nature of ulcerative colitis–certain individuals may not respond to S1P modulation–or from possible disease rebound phenomena. Notably, abrupt discontinuation of S1P modulators has led to severe disease reactivation in multiple sclerosis patients (as seen with fingolimod), and although such rebound in ulcerative colitis is not well characterized, the signal of “condition aggravated” calls attention to careful management around therapy interruptions. Overall, the safety signals observed are coherent with Etrasimod’s pharmacodynamic effects on the immune and cardiovascular systems and the pathophysiology of ulcerative colitis.

When comparing Etrasimod’s adverse event profile to other S1P receptor modulators, there are both parallels and improvements. Fingolimod (FTY720), the prototypical S1P modulator, is a non-selective agent that targets S1P_1,3,4,5 and was notorious for a number of safety liabilities (Chen et al., 2025). Fingolimod’s lack of receptor selectivity leads to off-target effects on S1P_3, which have been linked to bronchopulmonary toxicity and possibly to cardiac effects like hypertension. Most prominently, fingolimod causes a significant initial bradyarrhythmic effect–often requiring 6-hour first-dose cardiac monitoring–due to S1P_1 activation in cardiac tissue (Chen et al., 2025). It also has a very long half-life (∼6–9 days) and active metabolite, meaning adverse effects or immunosuppression can persist for weeks after stopping. Ozanimod, a second-generation modulator selective for S1P_1 and S1P_5, was designed to mitigate some of these issues. Head-to-head data in multiple sclerosis suggest that ozanimod has a milder cardiovascular profile than fingolimod (e.g., significantly lower risk of serious bradycardia or high-degree AV block) (Scott et al., 2016). This food–drug interaction warning is specific to ozanimod and not applicable to fingolimod or Etrasimod. Etrasimod, by contrast, was engineered with optimized S1P subtype selectivity and pharmacokinetics. By sparing S1P_3 and S1P_2, Etrasimod was expected to avoid S1P_3-mediated pulmonary and cardiotoxic effects while maintaining efficacy through S1P_1 (Chen et al., 2025). Indeed, Etrasimod’s receptor profile (S1P_1,4,5) is unique in also engaging S1P_4, which is expressed on lymphoid and immune cells–the relevance of S1P_4 modulation is still being elucidated but might contribute to immune cell positioning and possibly additional efficacy in gut-homing of lymphocytes. Importantly, Etrasimod has a short plasma half-life (∼30 h) (Are Made and List, 2025), meaning that drug levels (and lymphocyte counts) recover more quickly if the drug is stopped. This could translate to a lower risk of prolonged immunosuppression or severe rebound if therapy is interrupted, although robust evidence of this advantage will require further post-market observation. From a safety standpoint, early indications are that Etrasimod’s adverse event spectrum is broadly similar to ozanimod’s, and overall more favorable than fingolimod’s. For example, in clinical trials for ulcerative colitis, Etrasimod did not show an increased rate of serious cardiac outcomes versus placebo; only transient, first-dose bradycardia was noted (Vermeire et al., 2025).

It should be noted, however, that the absence of such events in trials could simply be due to limited exposure; long-term, real-world data are needed to determine if Etrasimod carries any risk of malignancy or other rare events. In terms of infection risk, Etrasimod appears comparable to other S1P modulators: slight increases in mostly mild infections have been noted (e.g., nasopharyngitis, sinusitis), but serious opportunistic infections remain rare (Vermeire et al., 2025). Nonetheless, all S1P receptor modulators share certain class effects and precautions: for example, bradycardia with initiation, risk of macular edema, hypertension, elevated hepatic enzymes, and lymphopenia with attendant infection risk (notably, herpes zoster reactivation) have been reported with fingolimod and also observed to some degree with ozanimod and Etrasimod (Chen et al., 2025). Therefore, rigorous patient monitoring and selection remain important across this drug class, even as refinements like those in Etrasimod have improved overall safety.

It should be noted that our findings are consistent with Guo et al., who also identified gastrointestinal, general, and eye disorders as key safety concerns for etrasimod, reinforcing the reliability of these signals (Guo et al., 2025). By including data through mid-2025 and a larger cohort (967 patients, 2,104 AEs), our study detected additional significant PTs, particularly within the investigations SOC. Unlike Guo et al., we conducted head-to-head disproportionality analyses against other S1P modulators (fingolimod and ozanimod), allowing a class-level safety comparison that suggests a potentially more favorable profile for certain lab abnormalities but no clear advantage for other AEs. Moreover, our sensitivity analysis, which assessed signals by drug suspicion level, confirmed that associations such as proctitis ulcerative were strongest when etrasimod was the primary suspect, enhancing signal robustness. Finally, our demographic profiling complements previous findings by providing a detailed view of outcomes and reporting characteristics, offering a more comprehensive understanding of etrasimod’s post-marketing safety.

It is important to acknowledge several limitations of this FAERS-based analysis. As a spontaneous reporting system, FAERS is subject to under-reporting, stimulated reporting for new drugs, and incomplete case information. Specifically, limitations include: (I) insufficient clinical details (e.g., dose, duration, comorbidities); (II) inability to estimate incidence rates due to missing denominator data; (III) potential confounding by indication or concomitant medications; and (IV) the relatively short time since Etrasimod’s approval, leading to a limited number of available reports.

The presence of a safety signal in FAERS does not establish causality but highlights disproportional reporting that requires further verification. Reporting frequency can also be influenced by a drug’s novelty or clinical attention. Despite these constraints, FAERS data mining remains a valuable tool for early detection of rare or unexpected adverse events and provides hypothesis-generating insights that warrant confirmation through future epidemiologic and mechanistic studies.

## 5 Conclusion

In conclusion, this FAERS-based pharmacovigilance study, building upon and extending recent literature, provides a detailed and updated characterization of the post-approval safety profile of Etrasimod. By leveraging a more extensive dataset, employing comparative and sensitivity analyses, our work confirms previously reported signals while offering novel insights into etrasimod’s relative standing among S1P modulators and the robustness of its AE associations.

## Data Availability

The datasets presented in this study can be found in online repositories. The names of the repository/repositories and accession number(s) can be found in the article/Supplementary Material.
