# Supplementary material for: A real-world pharmacovigilance study of FDA adverse event reporting system (FAERS) events for etrasimod
Source: Front Pharmacol. 2025 Nov 11;16:1693090. doi: 10.3389/fphar.2025.1693090 (PMC12644091; doi:10.3389/fphar.2025.1693090)
Supplement: Supplementary file 1 [file Supplementaryfile1.docx]

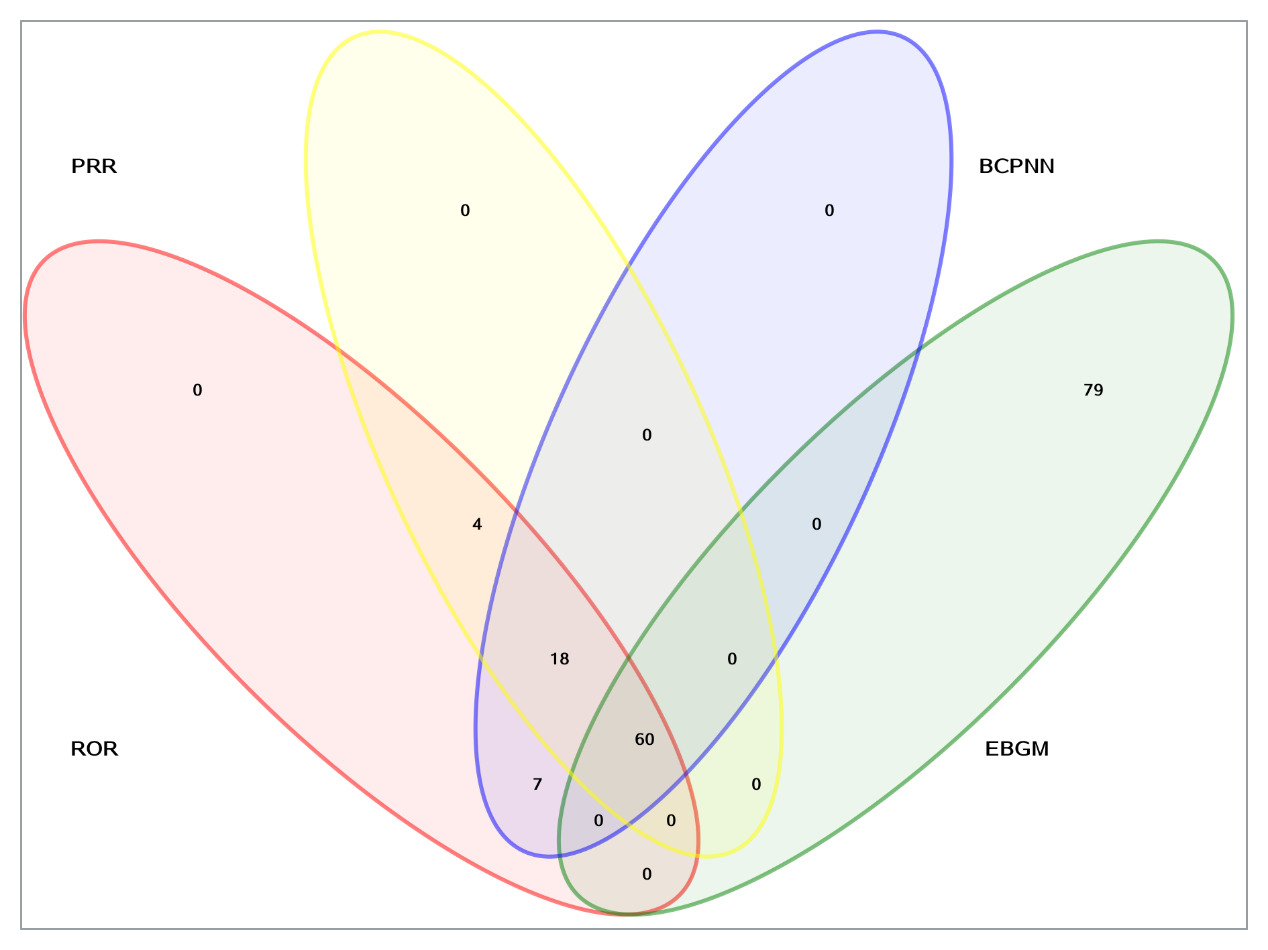


Figure S1. The Venn diagram of ROR, PRR, BCPNN, and MGPS (Not limited by the degree of suspicion).


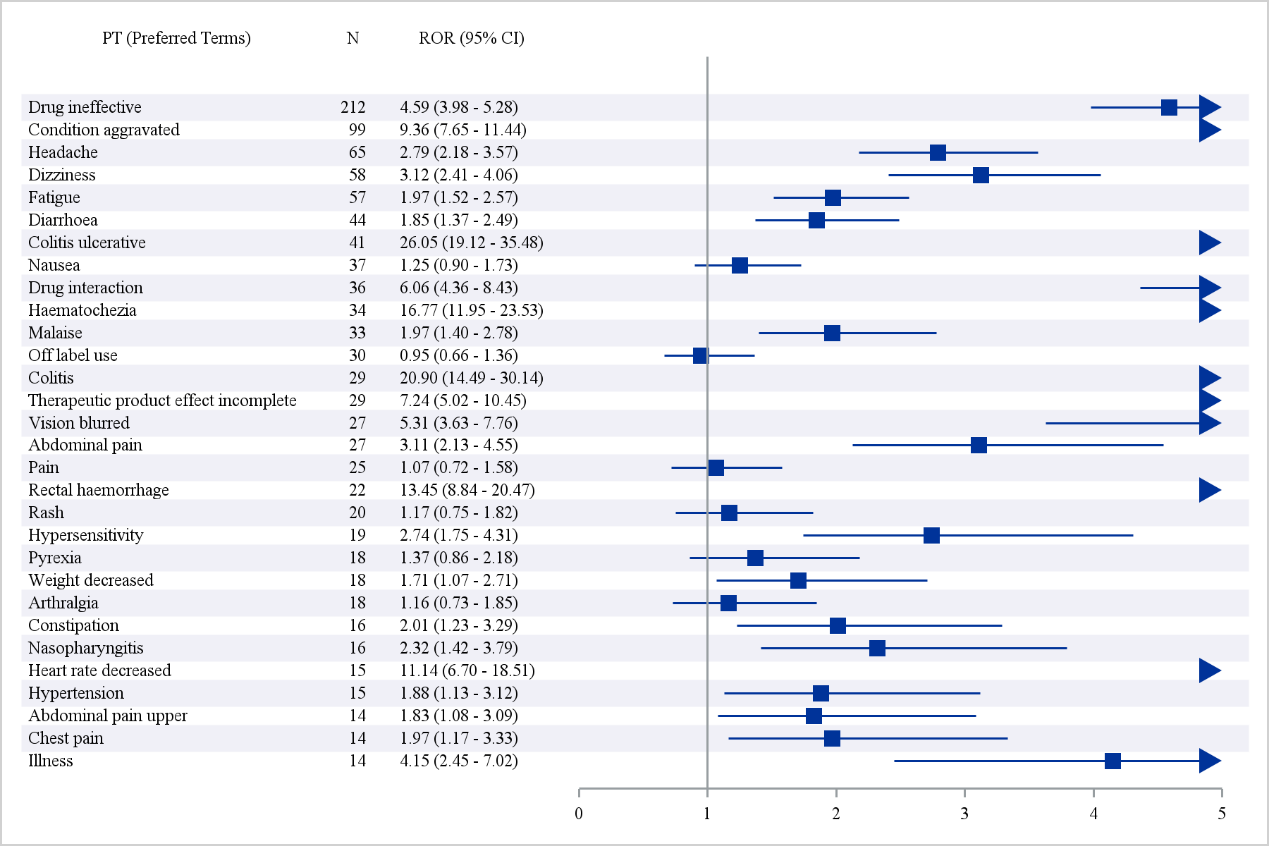


Figure S2. Forest plot for the frequency of the target drug (Not limited by the degree of suspicion).


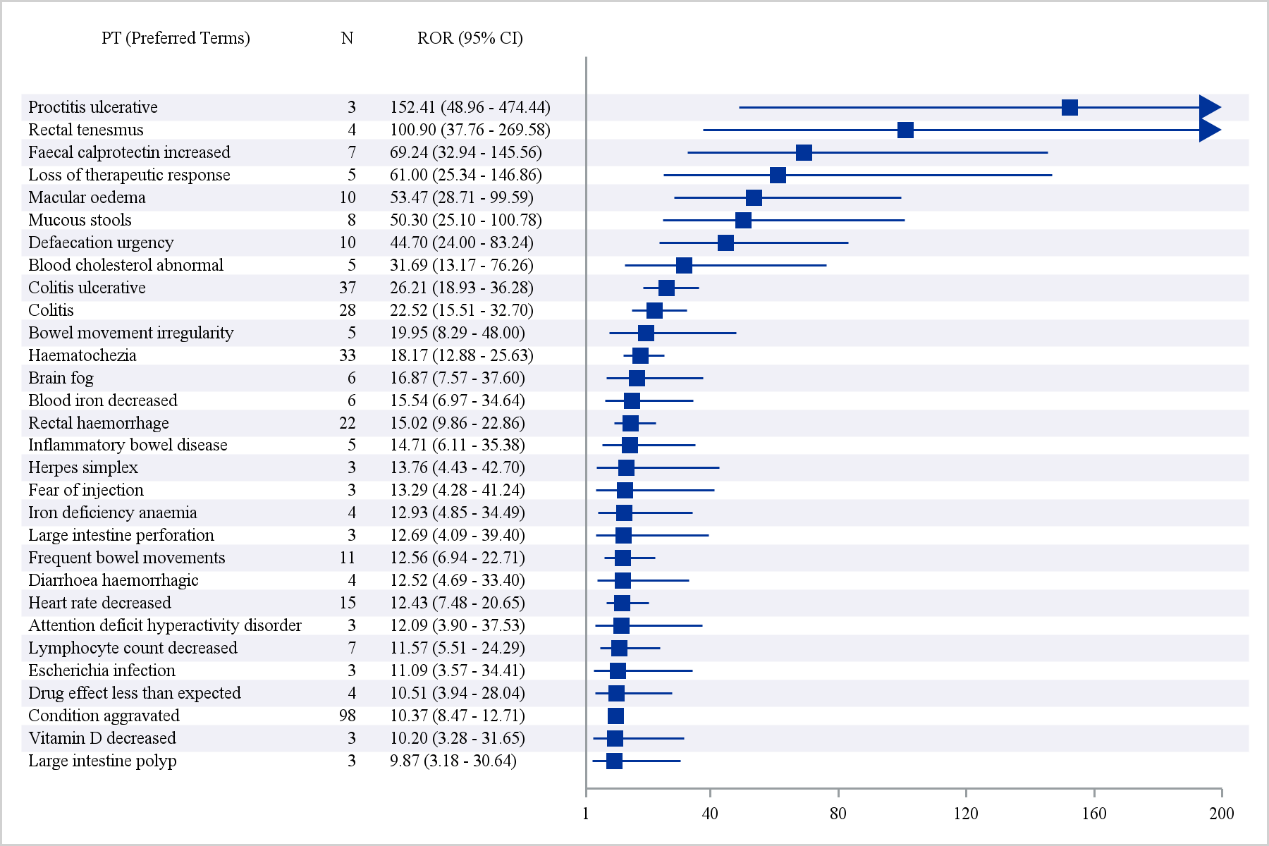


Figure S3. Forest plot for the signal strength for the target drug(Not limited by the degree of suspicion).

Table S1 Distribution of target drug–related adverse events by SOC (Not limited by the degree of suspicion).

| **System Organ Class (SOC)** | **Case number, n** | | **Case proportion, %** |
| --- | --- | --- | --- |
| General disorders and administration site conditions | 663 | 28.26 | |
| Gastrointestinal disorders | 483 | 20.59 | |
| Investigations | 205 | 8.74 | |
| Nervous system disorders | 197 | 8.40 | |
| Infections and infestations | 112 | 4.77 | |
| Skin and subcutaneous tissue disorders | 90 | 3.84 | |
| Eye disorders | 87 | 3.71 | |
| Musculoskeletal and connective tissue disorders | 72 | 3.07 | |
| Injury, poisoning and procedural complications | 69 | 2.94 | |
| Cardiac disorders | 53 | 2.26 | |
| Psychiatric disorders | 50 | 2.13 | |
| Respiratory, thoracic and mediastinal disorders | 44 | 1.88 | |
| Vascular disorders | 40 | 1.71 | |
| Immune system disorders | 38 | 1.62 | |
| Metabolism and nutrition disorders | 33 | 1.41 | |
| Blood and lymphatic system disorders | 25 | 1.07 | |
| Hepatobiliary disorders | 18 | 0.77 | |
| Surgical and medical procedures | 18 | 0.77 | |
| Renal and urinary disorders | 14 | 0.60 | |
| Neoplasms benign, malignant and unspecified (incl cysts and polyps) | 9 | 0.38 | |
| Reproductive system and breast disorders | 8 | 0.34 | |
| Ear and labyrinth disorders | 6 | 0.26 | |
| Endocrine disorders | 5 | 0.21 | |
| Congenital, familial and genetic disorders | 3 | 0.13 | |
| Social circumstances | 3 | 0.13 | |
| Product issues | 1 | 0.04 | |

Cases represent the number of adverse events classified under each System Organ Class (SOC).

Proportion (%) is calculated as the number of adverse events within each SOC divided by the total number of adverse events.

Table S2 Distribution of positive signals of adverse events of the target drug across different System Organ Classes (SOC) (Not limited by the degree of suspicion)

| **System Organ Class (SOC)** | **Case number, n** | | **Case proportion, %** | |
| --- | --- | --- | --- | --- |
| General disorders and administration site conditions | | 10 | 12.82 |  |
| Gastrointestinal disorders | | 27 | 34.62 |  |
| Investigations | | 12 | 15.38 |  |
| Nervous system disorders | | 4 | 5.13 |  |
| Infections and infestations | | 5 | 6.41 |  |
| Skin and subcutaneous tissue disorders | | 0 | 0.00 |  |
| Eye disorders | | 4 | 5.13 |  |
| Musculoskeletal and connective tissue disorders | | 1 | 1.28 |  |
| Injury, poisoning and procedural complications | | 1 | 1.28 |  |
| Cardiac disorders | | 2 | 2.56 |  |
| Psychiatric disorders | | 3 | 3.85 |  |
| Respiratory, thoracic and mediastinal disorders | | 0 | 0.00 |  |
| Vascular disorders | | 1 | 1.28 |  |
| Immune system disorders | | 2 | 2.56 |  |
| Metabolism and nutrition disorders | | 1 | 1.28 |  |
| Blood and lymphatic system disorders | | 1 | 1.28 |  |
| Hepatobiliary disorders | | 2 | 2.56 |  |
| Surgical and medical procedures | | 1 | 1.28 |  |
| Renal and urinary disorders | | 0 | 0.00 |  |
| Neoplasms benign, malignant and unspecified (incl cysts and polyps) | | 0 | 0.00 |  |
| Reproductive system and breast disorders | | 0 | 0.00 |  |
| Ear and labyrinth disorders | | 0 | 0.00 |  |
| Endocrine disorders | | 1 | 1.28 |  |
| Congenital, familial and genetic disorders | | 0 | 0.00 |  |
| Social circumstances | | 0 | 0.00 |  |
| Product issues | | 0 | 0.00 |  |
| Pregnancy, puerperium and perinatal conditions | | 0 | 0.00 |  |
| Total | | 78 | 100.00 |  |

The number of positive signals represents the count of Preferred Terms (PTs) within each SOC that were identified as signals using the specified method. This refers to the number of distinct PTs, not the number of PT occurrences.

The proportion (%) is calculated as the number of signals within each SOC divided by the total number of signals detected for the target drug.

Table S3 Top 30 Preferred Terms (PTs) ranked by frequency for the target drug (Not limited by the degree of suspicion).

| **Preferred Terms** | **Case number, n** | **ROR(95% CI)** | **PRR(Chi-Square)** | **IC(IC-2SD)** | **EBGM(EBGM05)** |
| --- | --- | --- | --- | --- | --- |
| Drug ineffective | 212 | 4.59(3.98-5.28) | 4.26(540.87) | 2.09(1.86) | 4.26(3.70) |
| Condition aggravated | 99 | 9.36(7.65-11.44) | 9.00(707.32) | 3.17(2.76) | 9.00(7.36) |
| Headache | 65 | 2.79(2.18-3.57) | 2.74(72.53) | 1.45(1.06) | 2.74(2.14) |
| Dizziness | 58 | 3.12(2.41-4.06) | 3.07(81.72) | 1.62(1.19) | 3.07(2.37) |
| Fatigue | 57 | 1.97(1.52-2.57) | 1.95(26.72) | 0.96(0.56) | 1.95(1.50) |
| Diarrhoea | 44 | 1.85(1.37-2.49) | 1.83(16.81) | 0.87(0.41) | 1.83(1.36) |
| Colitis ulcerative | 41 | 26.05(19.12-35.48) | 25.61(969.20) | 4.68(3.56) | 25.58(18.78) |
| Nausea | 37 | 1.25(0.90-1.73) | 1.25(1.81) | 0.32(-0.17) | 1.25(0.90) |
| Drug interaction | 36 | 6.06(4.36-8.43) | 5.99(149.89) | 2.58(1.92) | 5.99(4.31) |
| Haematochezia | 34 | 16.77(11.95-23.53) | 16.54(496.47) | 4.05(3.03) | 16.53(11.78) |
| Malaise | 33 | 1.97(1.40-2.78) | 1.96(15.58) | 0.97(0.43) | 1.96(1.39) |
| Off label use | 30 | 0.95(0.66-1.36) | 0.95(0.07) | -0.07(-0.59) | 0.95(0.66) |
| Colitis | 29 | 20.90(14.49-30.14) | 20.65(542.14) | 4.37(3.11) | 20.63(14.30) |
| Therapeutic product effect incomplete | 29 | 7.24(5.02-10.45) | 7.17(154.13) | 2.84(2.04) | 7.17(4.97) |
| Vision blurred | 27 | 5.31(3.63-7.76) | 5.26(93.30) | 2.39(1.64) | 5.26(3.60) |
| Abdominal pain | 27 | 3.11(2.13-4.55) | 3.09(38.24) | 1.63(0.97) | 3.09(2.11) |
| Pain | 25 | 1.07(0.72-1.58) | 1.06(0.10) | 0.09(-0.48) | 1.06(0.72) |
| Rectal haemorrhage | 22 | 13.45(8.84-20.47) | 13.33(251.06) | 3.74(2.51) | 13.33(8.76) |
| Rash | 20 | 1.17(0.75-1.82) | 1.17(0.50) | 0.23(-0.42) | 1.17(0.75) |
| Hypersensitivity | 19 | 2.74(1.75-4.31) | 2.73(20.90) | 1.45(0.68) | 2.73(1.74) |
| Pyrexia | 18 | 1.37(0.86-2.18) | 1.37(1.80) | 0.45(-0.24) | 1.37(0.86) |
| Weight decreased | 18 | 1.71(1.07-2.71) | 1.70(5.21) | 0.77(0.05) | 1.70(1.07) |
| Arthralgia | 18 | 1.16(0.73-1.85) | 1.16(0.41) | 0.22(-0.46) | 1.16(0.73) |
| Constipation | 16 | 2.01(1.23-3.29) | 2.00(8.08) | 1.00(0.22) | 2.00(1.23) |
| Nasopharyngitis | 16 | 2.32(1.42-3.79) | 2.31(11.93) | 1.21(0.40) | 2.31(1.41) |
| Heart rate decreased | 15 | 11.14(6.70-18.51) | 11.07(137.46) | 3.47(2.04) | 11.07(6.66) |
| Hypertension | 15 | 1.88(1.13-3.12) | 1.87(6.13) | 0.91(0.10) | 1.87(1.13) |
| Abdominal pain upper | 14 | 1.83(1.08-3.09) | 1.82(5.20) | 0.87(0.04) | 1.82(1.08) |
| Chest pain | 14 | 1.97(1.17-3.33) | 1.96(6.65) | 0.97(0.14) | 1.96(1.16) |
| Illness | 14 | 4.15(2.45-7.02) | 4.13(33.28) | 2.05(1.03) | 4.13(2.44) |

This table is ranked by frequency and presents only positive-signal Preferred Terms (PTs).

If the number of positive-signal PTs is fewer than 30, all detected PTs are presented.

Table S4 Top 30 Preferred Terms (PTs) ranked by signal strength for the target drug(Not limited by the degree of suspicion).

| **Preferred Terms** | **Case number, n** | **ROR(95% CI)** | **PRR(Chi-Square)** | **IC(IC-2SD)** | **EBGM(EBGM05)** |
| --- | --- | --- | --- | --- | --- |
| Adenosine deaminase decreased | 4 | 32570.7(7285.53-145611) | 32515.2(55736.9) | 13.77(0.68) | 13935.6(3117.18) |
| Large intestine erosion | 7 | 1990.88(920.48-4306.00) | 1984.94(12835.8) | 10.84(1.93) | 1835.61(848.69) |
| Ileocaecal resection | 4 | 384.69(143.15-1033.76) | 384.04(1504.47) | 8.56(1.00) | 378.10(140.70) |
| Ileal ulcer | 8 | 218.97(109.04-439.73) | 218.22(1714.50) | 7.76(2.15) | 216.30(107.71) |
| Proctitis ulcerative | 3 | 136.66(43.91-425.40) | 136.49(401.25) | 7.08(0.52) | 135.74(43.61) |
| Campylobacter infection | 4 | 132.76(49.65-354.97) | 132.53(519.35) | 7.04(0.98) | 131.82(49.30) |
| Rectal tenesmus | 4 | 90.47(33.87-241.70) | 90.32(352.03) | 6.49(0.96) | 89.99(33.69) |
| Cholangitis sclerosing | 6 | 79.16(35.48-176.62) | 78.96(460.38) | 6.30(1.61) | 78.71(35.28) |
| Biliary tract disorder | 4 | 76.94(28.81-205.48) | 76.81(298.35) | 6.26(0.95) | 76.57(28.67) |
| Faecal calprotectin increased | 7 | 62.08(29.53-130.48) | 61.89(418.33) | 5.95(1.82) | 61.74(29.37) |
| Loss of therapeutic response | 5 | 54.69(22.72-131.66) | 54.58(262.41) | 5.77(1.28) | 54.46(22.62) |
| Macular oedema | 10 | 47.93(25.74-89.26) | 47.73(456.68) | 5.57(2.31) | 47.64(25.58) |
| Mucous stools | 8 | 45.09(22.51-90.33) | 44.94(343.10) | 5.49(1.97) | 44.86(22.39) |
| Enteritis | 11 | 44.29(24.48-80.14) | 44.09(462.46) | 5.46(2.43) | 44.01(24.33) |
| Defaecation urgency | 10 | 40.07(21.52-74.60) | 39.90(378.66) | 5.32(2.26) | 39.84(21.39) |
| Appetite disorder | 7 | 29.52(14.05-62.02) | 29.44(192.08) | 4.88(1.67) | 29.40(14.00) |
| Drug specific antibody present | 7 | 28.66(13.64-60.20) | 28.57(186.07) | 4.84(1.66) | 28.54(13.59) |
| Blood cholesterol abnormal | 5 | 28.41(11.81-68.37) | 28.36(131.81) | 4.82(1.17) | 28.32(11.77) |
| Colitis ulcerative | 41 | 26.05(19.12-35.48) | 25.61(969.20) | 4.68(3.56) | 25.58(18.78) |
| Colitis | 29 | 20.90(14.49-30.14) | 20.65(542.14) | 4.37(3.11) | 20.63(14.30) |
| Anal abscess | 4 | 19.74(7.40-52.67) | 19.71(70.99) | 4.30(0.76) | 19.70(7.38) |
| Attention deficit hyperactivity disorder | 5 | 18.09(7.52-43.52) | 18.05(80.49) | 4.17(1.05) | 18.04(7.50) |
| Bowel movement irregularity | 5 | 17.89(7.44-43.03) | 17.85(79.49) | 4.16(1.05) | 17.84(7.42) |
| Haematochezia | 34 | 16.77(11.95-23.53) | 16.54(496.47) | 4.05(3.03) | 16.53(11.78) |
| Brain fog | 6 | 15.13(6.79-33.71) | 15.09(78.90) | 3.91(1.23) | 15.08(6.77) |
| Blood iron decreased | 6 | 13.93(6.25-31.05) | 13.90(71.80) | 3.80(1.20) | 13.89(6.23) |
| Rectal haemorrhage | 22 | 13.45(8.84-20.47) | 13.33(251.06) | 3.74(2.51) | 13.33(8.76) |
| Inflammatory bowel disease | 5 | 13.19(5.48-31.72) | 13.16(56.15) | 3.72(0.94) | 13.15(5.47) |
| Herpes simplex | 3 | 12.33(3.97-38.28) | 12.32(31.19) | 3.62(0.24) | 12.31(3.97) |
| Frequent bowel movements | 12 | 12.28(6.97-21.66) | 12.23(123.69) | 3.61(1.91) | 12.22(6.93) |

This table is ranked by frequency and presents only positive-signal Preferred Terms (PTs).

If the number of positive-signal PTs is fewer than 30, all detected PTs are presented.

Table S5 Comparative disproportionality analysis of key adverse event signal strengths between Etrasimod and other S1P modulators.

| Preferred Terms | Etrasimod ROR (95% CI) | Ozanimod ROR (95% CI) | Fingolimod ROR (95% CI) |
| --- | --- | --- | --- |
| Lymphocyte count decreased | 11.57 (5.51-24.29) | 21.40 (17.57-26.07) | 54.35 (52.35-56.42) |
| Macular oedema | 53.47 (28.71-99.59) | 16.48 (11.03-24.61) | 42.21 (39.27-45.38) |
| Proctitis ulcerative | 152.41 (48.96-474.44) | 26.12 (9.77-69.86) | — |
| Loss of therapeutic response | 61.00 (25.34-146.86) | 12.53 (6.26-25.09) | — |
| Defaecation urgency | 44.70 (24.00-83.24) | 9.16 (5.61-14.97) | — |
| Colitis ulcerative | 26.21 (18.93-36.28) | 20.49 (17.96-23.38) | — |
| Bowel movement irregularity | 19.95 (8.29-48.00) | 8.72 (5.41-14.03) | — |
| Heart rate decreased | 12.43 (7.48-20.65) | 8.51 (6.83-10.60) | — |
